# Fibrolipomatous Hamartoma of the Median Nerve with Macrodystrophia Lipomatosa

**DOI:** 10.7759/cureus.2293

**Published:** 2018-03-09

**Authors:** Muhammad Azeemuddin, Adeel A Waheed, Noman Khan, Raza Sayani, Anwar Ahmed

**Affiliations:** 1 Department of Radiology, The Aga Khan University, Karachi.

**Keywords:** fibrolipomatous hamartoma, neural fibrolipoma, macrodystrophia lipomatosa

## Abstract

Fibrolipomatous hamartoma (FLH) is a rare congenital condition that presents with a benign overgrowth of the bone and fibroadipose tissue termed as macrodystrophia lipomatosa (MDL). Although commonly seen in the median nerve, other peripheral nerves can be involved. Diagnosis can be made on magnetic resonance imaging (MRI) due to the characteristic coaxial cable appearance on axial images and the spaghetti appearance on sagittal images. Histology shows mature adipose and fibrous tissue infiltrating the epineural and perineural compartments. Multiple or debulking surgeries are often needed, with an emphasis on cosmetic aspects. We present one such case in which wide margin excision and sural nerve graft were carried out.

## Introduction

Fibrolipomatous hamartoma (FLH), also known as neural fibrolipoma (NFL), is a rare congenital condition caused by the benign growth of fibroadipose tissue surrounding nerve bundles. These are associated with bone overgrowth, resulting in macrodactyly in about one-third of the cases. This is termed as macrodystrophia lipomatosa (MDL) [1].

MDL is non-hereditary congenital macrodactyly characterized by the hyperplasia of the fibroadipose tissue and is usually accompanied by periosteal and endosteal new bone formation [2]. FLH is often seen in young patients. The median nerve is commonly affected although the involvement of other peripheral nerves is also reported in the medical literature [3]. Histology shows mature adipose and fibrous tissue infiltrating the epineural and perineural compartments. Affected nerves may show typical a pseudo-onion bulb and metaplastic new bone formation [1].

We present a case of MDL with FLH of the median nerve at the wrist.

## Case presentation

A 31-year-old man presented to our institution with complaints of a large swelling over the wrist and reduced mobility of the middle finger. The slowly growing mass had been noticed since birth. It was associated with intermittent pain and numbness, with a gradual increase in severity and duration over time. Because of the severity of symptoms, the patient had undergone carpal tunnel release at 19 years of age. Symptoms had improved significantly; however, they recurred within three years.

On examination, the patient was vitally stable. He had a palpable, nontender mass on the volar aspect of the right middle finger extending to the palm, mimicking hypertrophy of the right middle finger. Movement was restricted at the proximal and distal interphalangeal joints of the third finger. However, movement at the metacarpophalangeal joint was normal. A scar mark from the previous surgery was noted on the right wrist. There was marked swelling at the wrist; however, no sensory or motor deficit was noted on the wrist. The patient had no comorbid conditions and was admitted with a preliminary diagnosis of right middle finger tumor.

**Figure 1:**
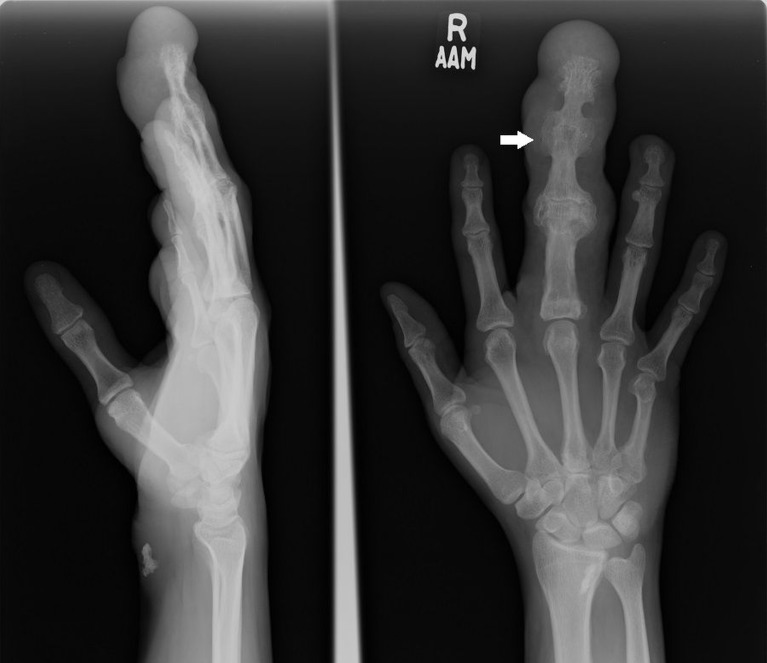
Right hand radiograph shows the lengthening of the middle finger (arrow) with a splaying and broadening of the middle phalanx due to secondary degenerative changes and diffuse soft tissue swelling.

A plain radiograph showed bony hypertrophy/overgrowth, resulting in a large right middle finger with a modeling deformity of the underlying bones (Figure 1).

**Figure 2:**
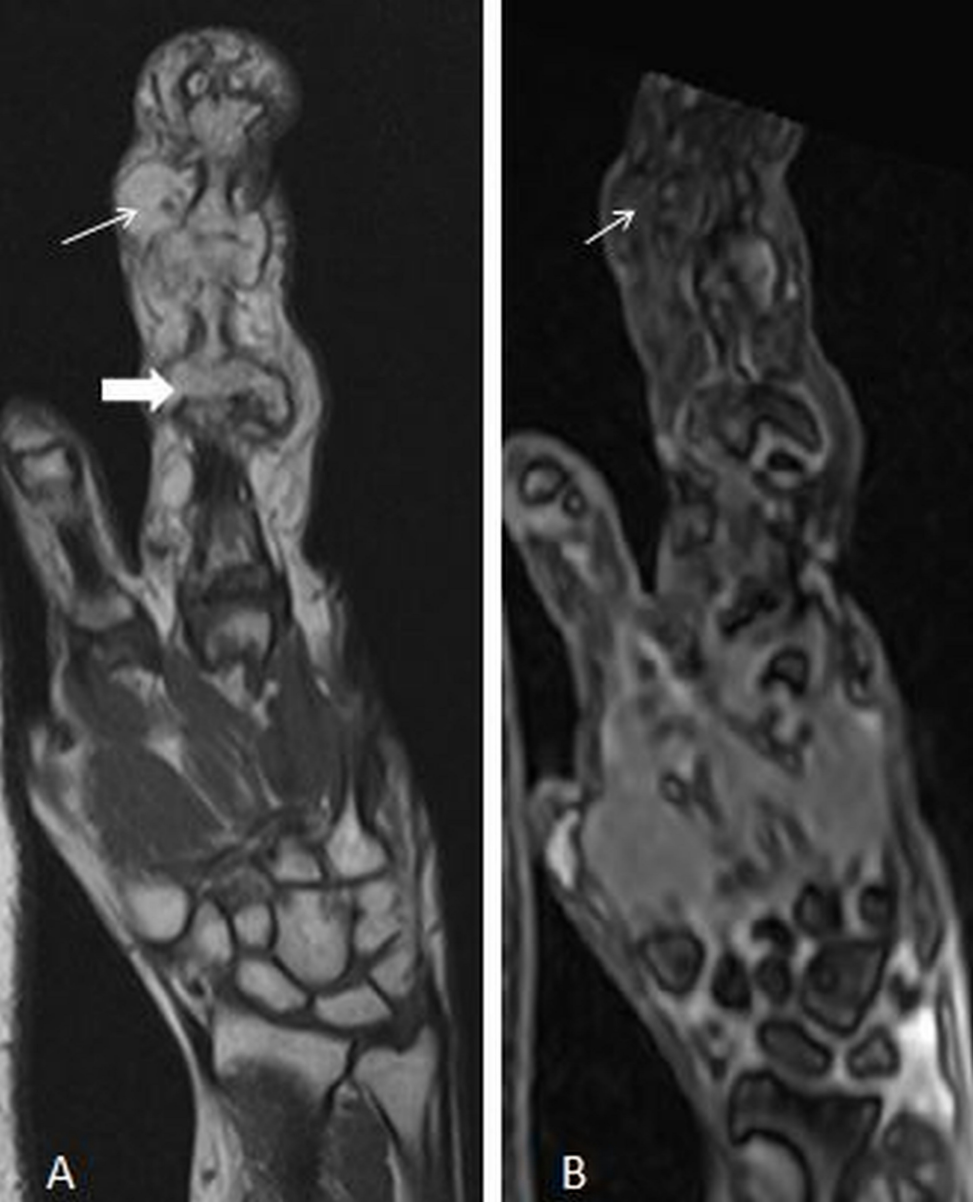
A) T1-weighted coronal image shows lipomatous hypertrophy of the third digit (thin arrow) with secondary osteoarthritic changes in the interphalangeal joints (thick arrow). B) T1 FS coronal image shows suppression of hypertrophied fat tissue in the third digit (thin arrow). Underlying marrow is not involved.

On magnetic resonance imaging (MRI), macrodactyly of the third digit was noted. Hypertrophic degenerative changes were seen in the interphalangeal joints with large osteophytes and loss of joint space. Fatty proliferation was also noted around the third digit. A sagittal T1W image showed lipomatous hypertrophy of the third digit with secondary osteoarthritic changes in interphalangeal joints (Figure 2).

**Figure 3:**
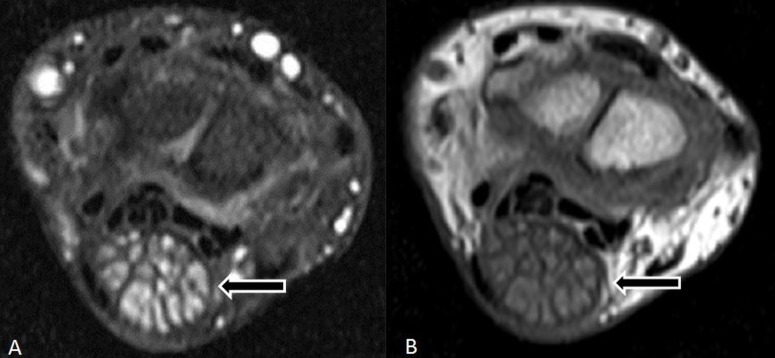
A) Axial T1-weighted FS + C image and B) Axial T2-weighted image of wrist shows significantly enlarged median nerve with hypertrophied nerve fascicles (arrow) stretching the flexor retinaculum, causing the carpal tunnel syndrome.

The median nerve appeared significantly enlarged with hypertrophied nerve fascicles stretching the flexor retinaculum and resulting in the carpal tunnel syndrome on axial T2W FS images. It appeared relatively hyperintense relative to muscles on T1-weighted images and isointense on T2-weighted images (Figure 3).

The overall appearance represented MDL of the right third digit with FLH of the median nerve. Under general anesthesia, the patient underwent wide margin excision of the right middle finger. In addition, a nerve graft of the sural nerve was applied to the median nerve. Intraop findings showed a right middle finger tumor involving the median nerve at the wrist. The tumor involved branches of the median nerve to the thumb, index, and ring finger. Specimens were sent for histopathology.

Post-procedure, the patient was discharged in a vitally stable condition. The patient developed surgical site infection and was treated for it. Short-term follow-up revealed the development of surgical site infection, which was successfully treated with broad-spectrum antibiotics. Follow-up at two months revealed partial regain of function and improved mobility.

The sample obtained during surgery was sent for a histopathologic examination, which confirmed the MRI findings. The lesion showed mature adipose tissue admixed with fibrous tissue, which dissected between and separated individual nerve bundles. Multiple nerve bundles were noted with perineural septation and pseudo-onion bulb formation along with concentric perineurial fibrous tissue.

## Discussion

The term MDL is thought to be first described by Mason [4]. FLH is usually located in the carpal tunnel and extends distally to the palm or fingers. The involvement of the proximal portion of the median nerve above the wrist is rare [5]. Although the common location is the median nerve or its branches, other peripheral nerves, such as radial nerve, ulnar nerve, nerves at the dorsal aspect of the foot, brachial plexus, and cranial nerves, may be involved [6].

MRI typically shows FLH with a high signal intensity of fat on both T1-weighted and T2-weighted images. There is a characteristic coaxial cable appearance on axial images and a spaghetti appearance on sagittal images [2]. A biopsy is often unnecessary, as magnetic resonance findings offer an accurate diagnosis [7].

FLH with or without MDL is an uncommon disease. Radiographic and MRI features may lead to accurate diagnosis and obviate the need for a biopsy. The main goal of management is symptomatic relief and cosmesis. Multiple or debulking surgeries are often adopted [8]. Cosmetic considerations are important, as total tumor resection cannot be achieved without sacrificing the affected nerve. In our case, a satisfactory surgical outcome was achieved with a wide margin excision and a sural nerve graft.

## Conclusions

FLH is a rare congenital condition that presents with a benign overgrowth of bone and fibroadipose tissue, termed as MDL. Diagnosis can be made on the basis of MRIs due to unique findings. Cosmetic considerations are important, as multiple surgeries are often required. We present one such case in which a satisfactory functional outcome was achieved with a wide margin excision and a sural nerve graft.
